# Urinary volatile metabolites of amygdala-kindled mice reveal novel biomarkers associated with temporal lobe epilepsy

**DOI:** 10.1038/s41598-019-46373-8

**Published:** 2019-07-22

**Authors:** Akiko Fujita, Manami Ota, Keiko Kato

**Affiliations:** Faculty of Life Sciences, Kyoto Sangyo University, Motoyama, Kamigamo, Kita-ku, Kyoto, Japan

**Keywords:** Biomarkers, Epilepsy

## Abstract

Epilepsy is a chronic neurological disorder affecting mammals, including humans. Uncontrolled epilepsy is associated with poor quality of life, accidents, and sudden death. In particular, temporal lobe epilepsy (TLE) is the most common type of pharmacoresistant epilepsy, which easily gets out of control in human adults. The aim of this study was to profile urinary volatile organic compounds (VOCs) in a mouse model of TLE using solid-phase microextraction (SPME) gas chromatography mass spectrometry (GC-MS). Thirteen urinary VOCs exhibited differential abundance between epileptic and control mice, and the corresponding areas under the receiver operating characteristic (ROC) curve were greater than 0.8. Principal component analysis (PCA) based on these 13 VOCs separated epileptic from sham operated-mice, suggesting that all these 13 VOCs are epilepsy biomarkers. Promax rotation and dendrogram analysis concordantly separated the 13 VOCs into three groups. Stepwise linear discriminant analysis extracted methanethiol; disulfide, dimethyl; and 2-butanone as predictors. Based on known metabolic systems, the results suggest that TLE induced by amygdala stimulation could affect both endogenous metabolites and the gut flora. Future work will elucidate the physiological meaning of the VOCs as end-products of metabolic networks and assess the impact of the metabolic background involved in development of TLE.

## Introduction

Epilepsy is a chronic disorder of the brain that affects approximately 50 million people of all ages worldwide. Approximately 30% of people with epilepsy are under inadequate control of their seizures and refractory to treatment with drugs^1,2^. Epilepsy is characterized by seizures, transient behaviors caused by disordered, synchronized, and rhythmic firings of neuronal groups in the brain, which propagate to regions connected with the first insult by neural circuits^3^. There are many forms of epilepsy, with multiple intracranial and extracranial causal factors and different natural histories. Patients of drug-resistant epilepsy receive alternative treatment through dietary manipulation, such as a ketogenic diet high in fat, with adequate amount of protein and low in carbohydrates: Such diet improves seizures especially in childhood^4,5^. Dietary manipulations seem to be useful in multiple seizure types. It has been suggested that extracranial metabolic changes affect the intracranial metabolic systems, leading to improvement of epileptic seizures. Conversely, it has also been suggested that epileptic seizures themselves and antiepileptic drugs lead to metabolic changes accompanied by changes in body weight^6^ and hormonal alterations^7^. Hence, if seizures affect extracranial metabolic systems, metabolites could be used as biomarkers of epilepsy. As patients suffering from epilepsy need care and support in their lives, the detection of metabolic changes through biomarkers would contribute to their safety and the prevention of insults.

Epilepsy also occurs naturally in other mammals^8^, such as rodents^9^, canines^10^, felines^11^, cattle^12^, goats^13^, horses^14^, and non-human primates^15^. Due to the lack of verbal communication, the initial discovery of the insults in animals often happens late in the course of the disease, when symptoms have become severe, leading to high lethality. Additionally, animals need to receive general anesthetization before medical screening, including electro-encephalography (EEG), which puts the animal under additional stress. Hence, extracranial metabolic products as biomarkers are needed also for epileptic animals.

Temporal lobe epilepsy is exhibited by half the patients with refractory epilepsy and mesial temporal lobe epilepsy (MTLE) includes foci in the amygdala, hippocampus, and surrounding cortex^16^. MTLE is characterized by hippocampal pathological signs including aberrant gene expression, morphological abnormalities^17^, seizures, and a high risk of comorbidity. One-half of patients with pharmacoresistant epilepsy have TLE, which is the most common type of epilepsy in adults^16^. Uncontrolled epilepsy worsens the quality of life, increases physical and psychiatric comorbidities, and imposes a heavy burden on patients, caregivers, and society. Therefore, a urinary biomarker could help patients avoid several issues and accidents. They could also be useful in animals, who are not able to communicate their symptoms to their owners. In the present study, we used an amygdala-kindled mouse model, in which conscious unrestrained mice received a biphasic square wave pulse into the basolateral amygdala once a day (for almost 3 weeks). This model was first established using rats in 1969^18^, then using canines^19^, felines^20^, apes^21^, and mice^22^. Animals that received amygdala-kindling stimulations show symptoms similar to those of human MTLE.

In this work, we focused on extracranial metabolites detected in urine and performed urinary volatile metabolic profiling of amygdala-kindled mice by gas chromatography - mass spectrometry (GC-MS) analysis, to develop novel biomarkers associated with temporal lobe epilepsy.

## Results

**Figure 1:**
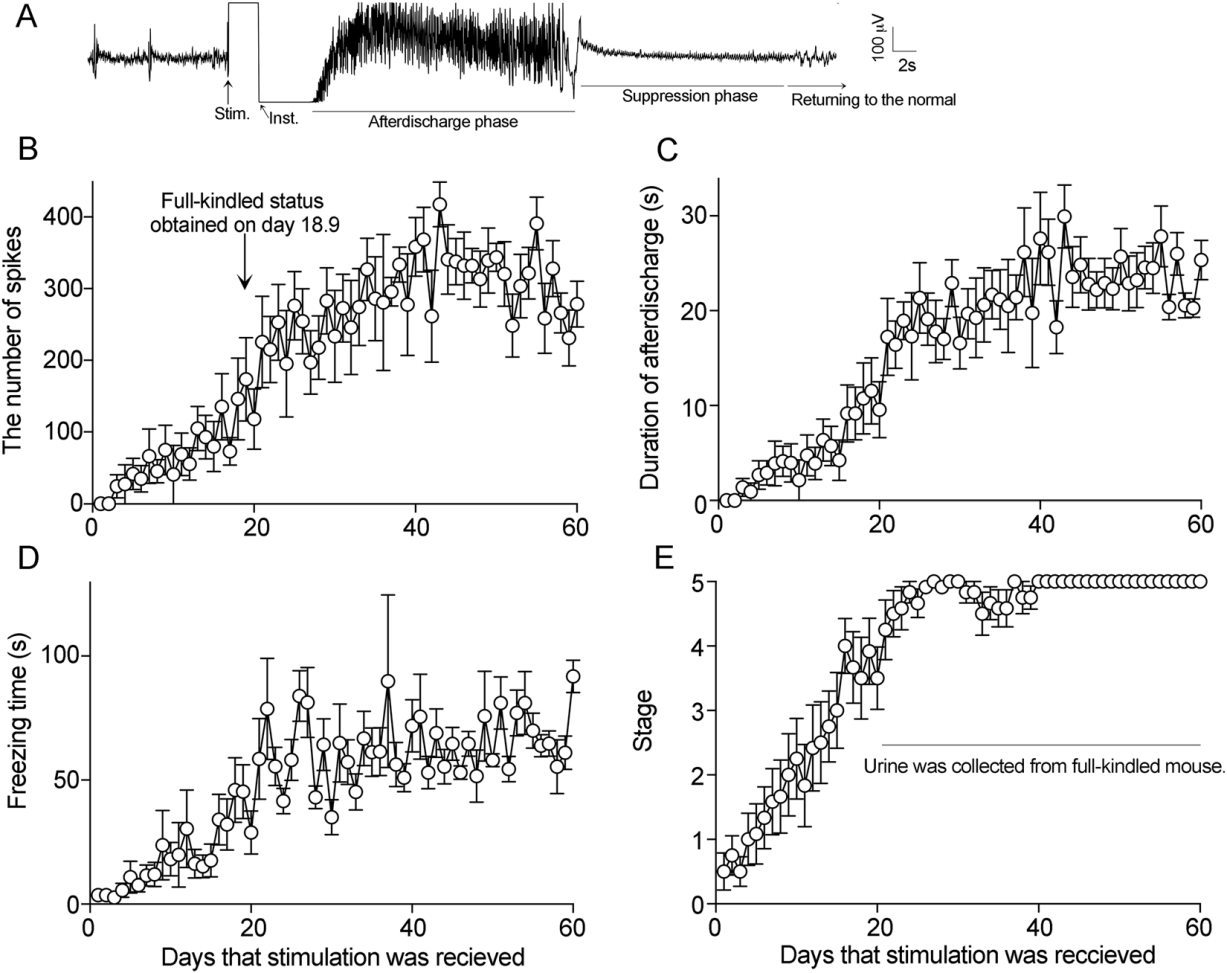
Preparation of amygdala-kindled mice and urine collection. (**A**) A typical EEG in a mouse during tonic-clonic seizures; (**B**) The number of spikes just after stimulation; (**C**) Duration of afterdischarge phase; (**D**) Duration of the suppression phase; (**E**) Stage: stage 1, mouth and facial movement; stage 2, forelimb clonus and afterdischarge duration greater than 5 s; stage 3, forelimb clonus and freezing duration greater than 15 s; stage 4, tonic clonic seizures and tail up; stage 5, falling over. Data are shown as mean ± S.E.M. Mice showing stage 5 symptoms were considered full-kindled. The arrow shows the mean time in days (18.9) when mice attained fully kindled seizures. Urine was collected from the day the mice reached full kindling to day 60.

Thirty-one mice including 16 epileptic and 15 sham-operated control mice were included in the study. Stimulation of the amygdala once a day increased the number of spikes and the duration of the afterdischage, and finally induced tonic-clonic seizures (Fig. 1). Urine was collected at age 13.5 to 18.5 weeks in amygdala kindled mice.

### Determination of the urinary volatile profiles of epileptic mice by SPME GC-MS

**Figure 2:**
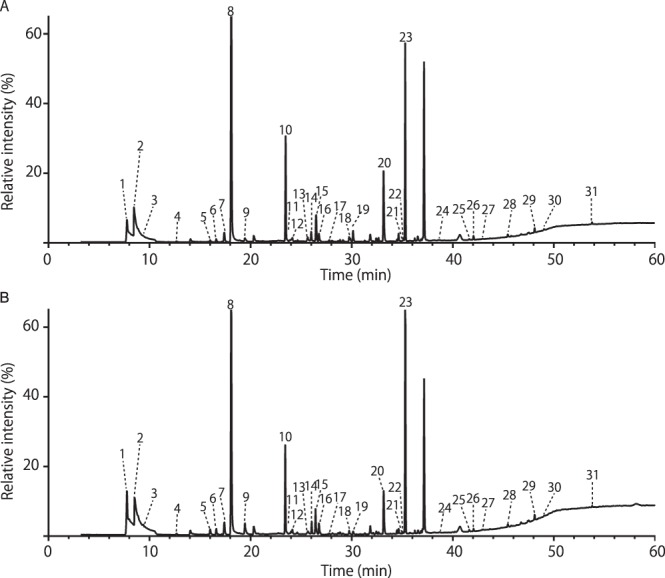
Typical GC-MS total ion chromatogram (TIC) of urinary volatile organic compounds (VOCs) in sham-operated and full-kindled mice. (**A**) Sham-operated mouse (**B**) Full-kindled mouse. The TICs were obtained from the analysis of the samples (200 µL) by HS-SPME (DVB/CAR/PDMS, 50/30 µm, 2 cm) and GC-MS equipped with an InertCap Pure-WAX column (60 m + 10 m pro-guard line and 2 m transfer line, 0.25 mm i.d., 0.5 um thick). The extraction temperature and time were respectively 45 °C and 60 min. Desorption was performed at 240 °C for 10 min. The injection was pulsed splitless (closed for 3 min). Temperature programming consisted of an initial temperature 40 °C for 10 min, followed by an increase of 5 °C/min to 240 °C, and a 10 min hold at the final temperature. Numbers indicate the following metabolites, which showed similarity indices above 85%: (1) carbon dioxide/carbamic acid, monoammonium salt/dl-alanyl-l-alanine; (2) trimethylamine; (3) methanethiol; (4) acetone; (5) 2-butanone; (6) butanal, 2-methyl-; (7) ethanol; (8) 2-hexenal, 2-ethyl-; (9) 2-pentanone; (10) 2-pentenal, 2,4,4-trimethyl-; (11) disulfide, dimethyl; (12) butanenitrile, 2-methyl-; (13) 3-penten-2-one; (14) 1-butanol; (15) RI1148; (16) methane, nitro-; (17) 2-heptanone; (18) 4-hepten-2-one, (E)-; (19) 5-oxohexanenitrile; (20) 2-acetyl-1-pyrroline; (21) dimethyl trisulfide; (22) 1-nitro-2-methyl propene; (23) 3,4-dehydro-exo-brevicomin; (24) benzaldehyde; (25) butanoic acid, 3-methyl-; (26) acetophenone; (27) 2,3,5-trithiahexane; (28) benzenamine, 3-methyl-; (29) hexanoic acid, 2-ethyl-; (30) 2-acetylpyrrole; (31) formamide, N-phenyl-.

Typical SPME GC-MS TIC chromatograms of urine samples from an epileptic and a sham-operated mice are shown in Fig. 2, and indicate that very similar VOC profiles were obtained from the two groups of samples.

One hundred thirty-five metabolites were detected in both epileptic and sham-operated mice using GC-MS (Shimadzu QP-2010 Ultra and TQ-8040) (Table S1), including a variety of chemical structures, such as aldehyde, ketones, nitrogen compounds, terpenes, acids, alcohols, benzene derivatives, furan, sulphur, etc. XCMS extracted 24 VOCs showing differential fragment ion *m/z* values between the two groups of samples (Table 1). Next, the fragment ion *m/z* values of the VOCs with the largest area within each fragmentation pattern in the 24 VOCs were selected to compare their absolute area values between the two groups of samples, resulting in fifteen VOCs as potential biomarkers (*p*-value < 0.05, marked with a star in column 16, Table 1). In this study, the data were not corrected for the creatinine content. Compounds for which the retention index (RI) of the detected compound did not match literature data or authentic compounds were considered as unknown and named using their RI.

**Table 1:** Identification of urinary volatile organic compounds (VOCs) by XCMS, showing differential levels between full-kindled and sham-operated mice.

	Mainly observed *m/z*	Quantified ion (*m/z*)^a^	RI^b^ (polar)	RI^c^ (non-polar)	Compound	SI^d^	Chemical class	CAS No	Chemical formula	Relative values of kindling mice to sham mice (fold)	Absolute area of *m/z*				*p*-value^e^
											Kindling (n = 11)		Sham (n = 10)		
											Average (×10^3^)	S.E.M. (×10^3^)	Average (×10^3^)	S.E.M. (×10^3^)	
1	58,59,42	58		n.d.	Trimethylamine^†^	97	Tertiary aliphatic amine	75-50-3	C3H9N	0.54	6612.2	1002.4	12245	1773.5	0.0037**
2	45,47,48	47		n.d.	Methanethiol^†^	96	Thiol	74-93-1	CH4S	0.6162	47.184	4.8305	76.577	6.0528	0.0028**
3	43,57,72	43	900	n.d.	2-Butanone^†^	98	Ketone	78-93-3	C4H8O	1.3098	876.52	44.931	669.19	56.75	0.0127*
4	41,43,86	43	996	n.d.	2-Pentanone^†^	98	Ketone	107-87-9	C5H10O	1.9853	3090.9	364.01	1556.9	282.28	0.0028**
5	45,79,94	94	1083	n.d.	Disulfide, dimethyl^†^	96	Disulfide	624-92-0	C2H6S2	0.5395	35.44	5.1244	65.693	5.13	0.0008***
6	69,84,71	69	1130	n.d.	3-Penten, 2-one^†^	94	Ketone	3102-33-8	C5H8O	0.763	261.33	38.193	342.51	33.607	0.0986
7	126,57,55	57	1148		RI1148^f^	85				1.0063	644.72	74.86	640.71	71.755	0.9725
8	30,46,61	30	1149	n.d.	Methane, nitro-^#^	94	Nitro	75-52-5	CH3NO2	0.6607	509.6	81.819	771.34	68.185	0.0079**
9	43,58,71	43	1179	n.d.	2-Heptanone^†^	95	Ketone	110-43-0	C7H14O	0.5543	155.84	21.563	281.17	26.046	0.0021**
10	55,69,98	69	1213		RI1213^f^	94	Ketone	6672-30-6	C6H10O	0.7969	59.424	8.7333	74.573	9.1587	0.2512
11	43,41,94	43	1235		RI1227^f^	90			C7H12O	0.3093	115.94	41.742	374.78	45.253	0.0008***
12	43,41,94	43	1242		RI1237^f^	90			C7H12O	0.6465	941.21	212.59	1455.8	227.24	0.1321
13	57,69,97	57	1291		RI1291^f^	89				1.171	212.27	23.647	181.27	21.291	0.3494
14	41,43,71	43	1310		RI1310^f^					0.8617	315.3	32.427	365.89	30.966	0.223
15	43,41,42	43	1334	n.d.	2-Acetyl-1-pyrroline^#^	91	Ketone/Pyrroline	85213-22-5	C6H9NO	0.629	4526.3	503.67	7196	510.72	0.0048**
16	126,79 45	126	1392	n.d.	Dimethyl trisulfide^†^	91	Trisulfide	3658-80-8	C2H6S3	0.5121	6.6292	1.5737	12.945	1.9504	0.0159*
17	39,53,84	39	1400	n.d.	1-Nitro-2-methyl propene^†^	89	Nitro	1606-30-0	C4H7NO2	1.2223	16.529	1.3814	13.523	0.8371	0.0845
18	43,111,95	43	1406	1024	3,4-dehydro-*exo*-brevicomin ^##^	88	Bridged bicyclo	62255-25-8	C9H14O2	1.3886	8958.9	530.4	6451.5	863.97	0.0357*
19	41,42,55	55	1449		RI1449^f^					0.638	255.22	32.12	400.02	31.662	0.0079**
20	41,43,60	60	1642	n.d.	Butanoic acid, 3-methyl-^†^	96	Fatty acid	503-74-2	C5H10O2	1.5452	175.01	32.423	113.26	26.693	0.1321
21	105,77,120	105	1662	n.d.	Acetophenone^†^	96	Ketone	98-86-2	C8H8O	0.5489	119.65	15.353	218.01	25.385	0.0062**
22	45,61,140	61	1680	n.d.	2,3,5-Trithiahexane^#^	96	Disulfide	42474-44-2	C3H8S3	0.23	7.8006	4.3366	33.917	5.2601	0.0006***
23	66,94,109	94	1964	n.d.	2-acetylpyrrole^†^	94	Pyrrole	1072-83-9	C6H7NO	0.6947	52.332	3.998	75.329	4.824	0.0028**
24	93,66,121	93	2184	1164	Formamide, N-phenyl-^##^	92	Amide	103-70-8	C7H7NO	0.5554	77.19	10.133	138.98	30.951	0.1145
										**Relative value**	**Average**	**S.E.M**.	**Average**	**S.E.M**.	***p*** **-value** ^e^
					Creatinine (mg/dL)^g^					1.1995	72.88	3.932	60.76	4.912	0.0687

The urine of male mice was collected daily since they acquired epileptic seizures. The NIST 14 standard reference database^60^ tentatively identified VOCs from peaks of total ion current (TIC) chromatogram as described in Table S1. VOCs in amygdala-stimulated kindled and sham-operated mice were analyzed with XCMS, resulting in 24 VOCs. Quantified ions were used to calculate the peak areas of the VOCs.

^a^The area of an ion peak was used for quantification of urinary VOCs.

^b^Retention indices of VOCs by the InertCap PureWAX column.

^c^Retention indices of VOCs by the DB-1 column (n.d., “not detected”).

^d^The similarity indices (SI) show the similarity with the mass spectrum from the NIST 14 standard reference database. VOCs with SI greater than 85 were described.

^e^Mann Whitney *U*-test (two-tailed).

^f^There was no identical pattern of *m/z* with the authentic compounds and the retention index of the literature data exhibited in the NIST library, while the maximum similarity index with the MS spectrum was above 85%.

^g^Urinary creatinine concentrations were determined by LabAssay^TM^ Creatinine colorimetry kit (Wako Pure Chemical industries, Ltd. Osaka), based on the Jaffe method^59^.

^†^VOCs were confirmed by identification with commercial standard references.

^#, ##^Hashes indicate that the retention index of the VOCs calculated with retention time in the polar (InertCap PureWAX) ^#^and non-polar (DB-1) ^##^columns were identical with the literature data, respectively.

### Receiver operating characteristic (ROC) curves

The ROC curves of fifteen VOCs were graphed to identify the optimal cut-off value using the absolute area of each ion peak shown in Table 1 (data not shown). The area under the ROC curve (AUC), and sensitivity, specificity, and accuracy at the cut-off point were calculated to evaluate the discriminatory power of the potential biomarkers in tonic-clonic seizures (Table 2). The statistical analysis of each individual compound significantly different between the groups revealed excellent predictive power, as shown by the AUC values: the AUC of disulfide, dimethyl was 0.9091 (95% confidence interval (CI) 0.7858 to 1.032), with an accuracy of 0.8571 (sensitivity = 0.8182, specificity = 0.9000). The AUC of RI1227 was 0.9091 (95% CI 0.7532 to 1.065) with an accuracy of 0.9048 (sensitivity = 0.9091, specificity = 0.9000). The AUC for 2,3,5-trithiahexane (disulfide, methyl (methylthio) methyl) was 0.9182 (95% CI 0.7635 to 1.073) with an accuracy of 0.9524 (sensitivity = 0.9091, specificity = 1.0000). On the other hand, the sensitivity for 2-butanone was 1, showing no false negatives, while the specificity for 2,3,5-Trithiahexane was 1, showing no false positives. The AUCs for 14 VOCs (the exception being 3,4-dehydro-exo-brevicomin (7-Exo-ethyl-5-methyl-6,8-dioxabicyclo[3.2.1]oct-3-ene)) were greater than 0.8, indicating high potential as prospective epilepsy biomarkers. Additionally, 11 out of 15 species of VOCs have been detected in human specimens (indicated by a hash in Table 2, http://www.hmdb.ca), the exceptions being methane, nitro-, 3,4-dehydro-exo-brevicomin, RI1227, and RI1449.

**Table 2:** ROC evaluations of urinary organic compounds (VOCs) showing differential levels between kindled (n = 11) and sham-operated (n = 10) mice.

NO^a^	VOCs	Cut off	Sensitivity	Specificity	Accuracy	AUC	p-value	95% confidence interval	http://www.hmdb.ca ^#^
1	Trimethylamine	<7.998e + 006	0.8182	0.9000	0.8571	0.8636	0.0049	0.6916 to 1.036	HMDB0000906
2	Methanethiol	<61264	0.8182	0.7000	0.7619	0.8727	0.0039	0.7249 to 1.021	HMDB0003227
3	2-Butanone	>597119	1.0000	0.6000	0.8095	0.8182	0.0137	0.6279 to 1.008	HMDB0000474
4	2-Pentanone	>1.792e + 006	0.9091	0.8000	0.8571	0.8727	0.0039	0.7046 to 1.041	HMDB0034235
5	Disulfide, dimethyl	<50680	0.8182	0.9000	0.8571	0.9091	0.0015	0.7858 to 1.032	HMDB0005879
8	Methane, nitro-	<692185	0.9091	0.6000	0.7619	0.8364	0.0092	0.6535 to 1.019	n.d.
9	2-Heptanone	<197443	0.8182	0.9000	0.8571	0.8818	0.0031	0.7319 to 1.032	HMDB0003671
11	RI1227	<232964	0.9091	0.9000	0.9048	0.9091	0.0015	0.7532 to 1.065	n.d.
15	2-Acetyl-1-pyrroline	<6.068e + 006	0.9091	0.8000	0.8571	0.8545	0.006	0.6865 to 1.023	HMDB0031308
16	Dimethyl trisulfide	<10507	0.8182	0.6000	0.7143	0.8091	0.0167	0.6231 to 0.9951	HMDB0013780
18	3,4-dehydro-*exo*-brevicomin	>7.457e + 006	0.8182	0.7000	0.7619	0.7727	0.0346	0.5608 to 0.9847	n.d.
19	RI1449	<325047	0.8182	0.8000	0.8095	0.8364	0.0092	0.6595 to 1.013	n.d.
21	Acetophenone	<165962	0.9091	0.8000	0.8571	0.8455	0.0075	0.6607 to 1.030	HMDB0033910
22	2,3,5-Trithiahexane	<7757	0.9091	1.0000	0.9524	0.9182	0.0012	0.7635 to 1.073	HMDB0031875
23	2-acetylpyrrole	<62510	0.9091	0.8000	0.8571	0.8727	0.0039	0.7153 to 1.030	HMDB0035882

The absolute values of fifteen VOCs showing significant differences between kindled and sham-operated mice (*p* < 0.05) as shown in Table 1 were evaluated by ROC analysis.

^a^Number described in Table 1. 3,4-dehydro-exo-brevicomin showed a fair ROC curve (AUC < 0.8); disulfide, dimethyl, RI1227, and 2,3,5-trithiahexane showed excellent curves (AUC > 0.9); the other 11 VOCs showed good ROC curves (0.8 < AUC < 0.9).

^#^Compounds that have been detected in human specimens, such as urine, faeces, and blood (http://www.hmdb.ca). n.d. “not described”.

Next, as 15 VOCs is a large numbers of biomarkers, we proceeded to narrow their number. Specifically, we tried to reduce the number of variables using principal component analysis (PCA), classify the VOCs by producing a dendrogram, and narrow their number using linear discriminant analysis.

### Principal component analysis and dendrogram analysis

**Figure 3:**
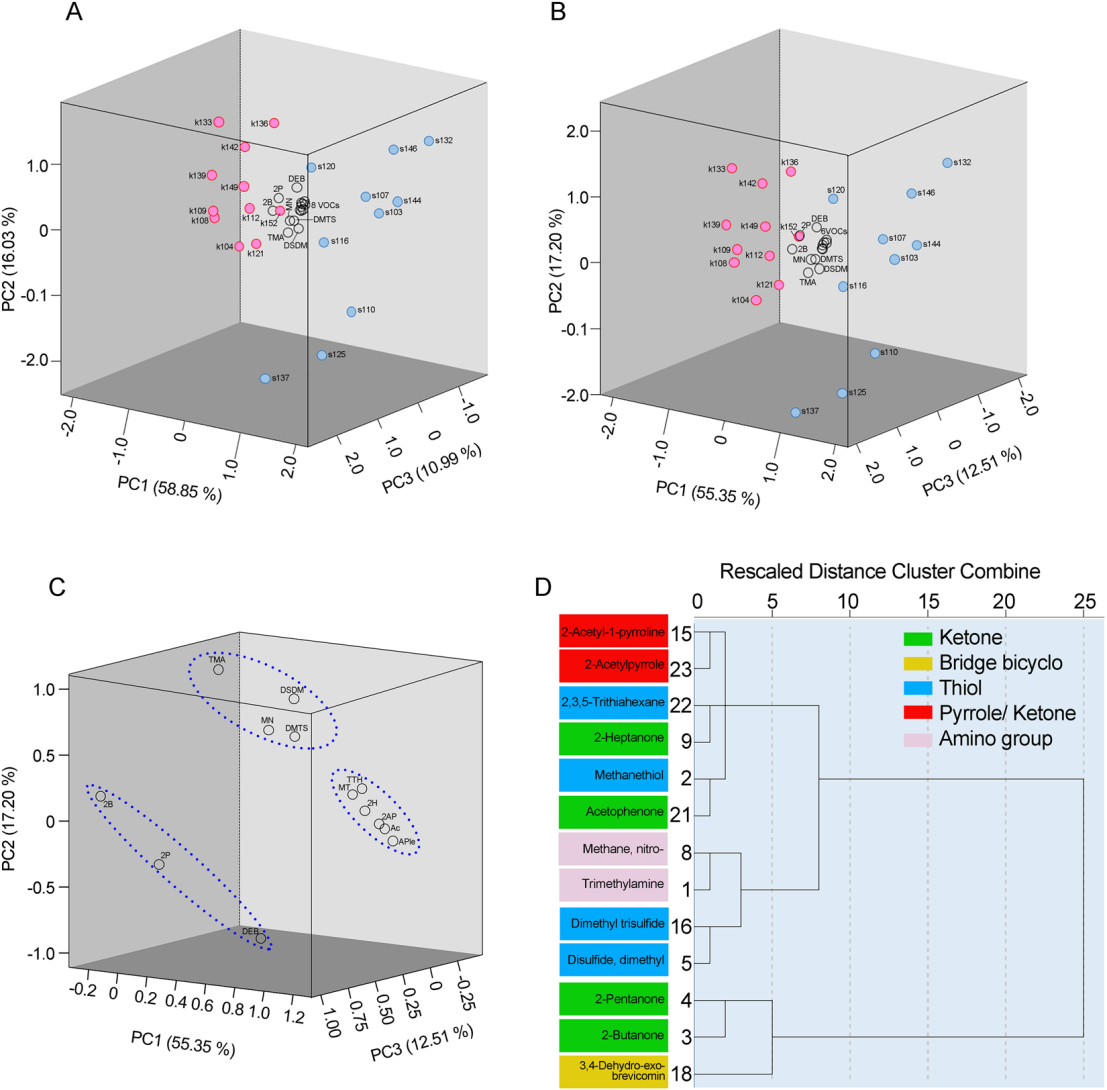
Evaluation of VOCs related with kindled seizures using principal component analysis (PCA). The principal component analysis (PCA) scores plot was derived from absolute values of 15 VOCs (**A**) and 13 VOCs (**B**) in the urine of amygdala-kindled and sham-operated mice. PC1 to PC3 scores of kindled mice (red circles), sham operated mice (blue circles), and PC1 to PC3 standardized scoring coefficients (black circles) in the principal component score coefficient matrix of each VOC were represented as the X-, Y-, and Z-axis, respectively. Promax rotation with Kaiser normalization was applied to the PCA with 13 VOCs, and components 1 to 3 of the pattern matrix of each VOC were represented as the X-, Y-, and Z-axis, respectively (**C**). A dendrogram of 13 VOCs was derived with the Ward method from the component scores 1 to 6 of each VOC (**D**). The vertical axis shows the number described in Table 1. Abbreviations: 2-acetyl-pyrroline, 2AP; 2-acetylpyrrole, APle; 2-butanone, 2B; 2-heptanone, 2H; 2-pentanone, 2P; acetophenone, Ac; 3,4-dehydro-exo-brevicomin, DEB; dimethyl trisulfide, DMTS; disulfide, dimethyl, DSDM; methane, nitro-, MN; methanethiol, MT; trimethylamine, TMA; 2,3,5-trithiahexane, TTH. The 6 VOCs include 2AP, 2H, Ac, APle, and MT, TTH in B; the 8 VOCs include the 6 VOCs in B and RI1227 and RI1449 in A.

The 15 VOCs differentially expressed in the urine of kindled vs. sham-operated mice (Table 1) and the 13 VOCs obtained after removing RI 1227 and RI 1449 (unknown compounds not found in human specimens) were analyzed by PCA using the absolute area of each ion peak. A separation trend was revealed in the three-dimensional PCA score plot using all 15 potential biomarker VOCs. The percentage of variance explained by the first three principal components was: PC1, 58.85% and 55.35% using 15 and 13 VOCs, respectively; PC2: 16.03% and 17.20%; PC3: 10.99% and 12.51%. Cumulatively, the first three components explained 85.87% and 85.06% of the variance, respectively. The principal component scores of kindled mice (Fig. 3A,B, red circles) are clearly separated from those of sham-operated mice (blue circles). This suggests that the urinary VOCs we identified are indeed able to distinguish kindled from sham-operated mice. The loadings of the 15 or 13 VOCs on the first three PCs are also shown in Fig. 3A,B (transparent circles), and are concentrated near the origin of the axes.

As the VOCs loadings of the 13 known VOCs were concentrated near the origin in the PCA scores plot, we applied promax rotation (kappa = 4) with Kaiser normalization, which converged in 5 iterations. After the rotation, high positive loadings on the first component (and low loadings on the second and third) were found for 2-acetylpyrrole (1.031), 2-acetyl-pyrroline (0.994), acetophenone (0.915), 2-heptanone (0.904), 2,3,5-trithiahexane (0.846), and methanethiol (0.804); while high positive loadings on the second component (and low loadings on the first and third) were found for trimethylamine (methylamine, *N*, *N*-dimethyl-) (0.990), disulfide, dimethyl (0.722), methane, nitro- (0.623), and dimethyl trisulfide (0.542); finally high positive loadings on the third component (and low loadings on the first and second) characterized 2-butanone (0.929), 2-pentanone (0.863), while the 1st, 2nd, and 3rd components of 3,4-dehydro-exo-brevicomin were 0.306, −0.991, and 0.177, respectively. Based on these loading scores, the VOCs could be divided into three groups (Fig. 3C). To confirm such grouping, we performed hierarchical clustering analysis with the scores of six – dimensional components extracted from the correlation matrix of the 13 VOCs in the PCA, leading to 3 groups of VOCs (Fig. 3D), which turned out to be identical to those found by promax rotation.

### Linear discriminant analysis

**Figure 4:**
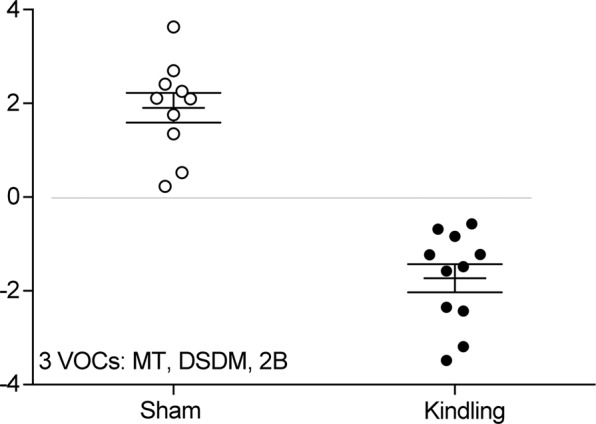
Scatter diagram of the stepwise linear discriminant analysis for determining VOCs associated with kindled seizures. Stepwise linear discriminant analysis with Wilk’s lambda was applied to the thirteen VOCs. F-value probabilities less than 0.05 led to retention and greater than 0.10 to deletion. The standardized canonical discriminant function coefficients were 0.772 (methanethiol), −0.882 (2-butanone), and 0.677 (disulfide, dimethyl) (eigenvalue, 3.650; canonical correlation, 0.886; Wilks’ lambda, 0.215; chi squared, 26.897, df (degrees of freedom), 3, *p* = 0.000006).

Finally, linear discriminant analysis was performed, as the Box’s M test suggested homogeneity of the covariance matrices (F (6, 2540) = 0.207, *p* = 0.975). The 13 known VOCs were used in the analysis and the stepwise method using Wilk’s lambda was applied to automatically select the best variables, with maximum value of the *F* probability for retention set at 0.05 and minimum for deletion at 0.10, resulting in an eigenvalue of 3.650 and canonical correlation of 0.886 (chi squared = 26.897, degrees of freedom (df) = 3, *p* = 6e-6). The standardized canonical discriminant function coefficients were 0.772 for methanethiol, −0.882 for 2-butanone, and 0.677 for disulfide, dimethyl. The discriminant function found is expressed by:

$$\begin{array}{rcl}{\rm{Di}} & = & -0.53887155117\\  &  & +\,0.00004396261\ast [{\rm{methanethiol}}]\\  &  & -\,0.00000537535\ast [2 \mbox{-} \mathrm{butanone}]\\  &  & +\,0.00004072790\ast [{\rm{disulfide}},{\rm{dimethyl}}]\end{array}$$

where the square brackets represent the absolute area of ion peak *m/z* for each VOC. Using this function, the discriminant scores of kindled (black circles) and sham-operated (white circles) mice were calculated and are shown in Fig. 4.

Finally, mice suffering from epilepsy were distinguished from controls by using the best biomarkers, i.e. methanethiol, disulfide, dimethyl, and 2-butanone, in which 100% of original grouped cases was correctly classified and 95.2% of cross-validated grouped cases was correctly classified (Fig. 4). Notably, each of these three VOCs belonged to a different group among those derived from the promax rotation (Fig. 3C) and the dendrogram analysis (Fig. 3D).

## Discussion

**Figure 5:**
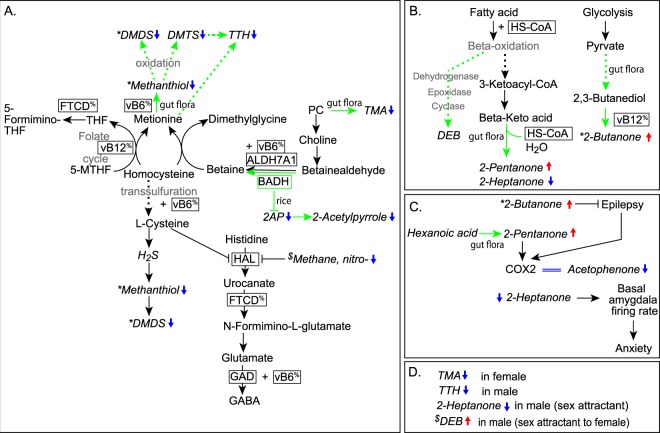
Metabolic networks (**A**,**B**) and representative physiological systems (C and D) containing the VOCs associated to kindled seizures. Transsulfation^30,32,35^, methionine-homocystaine cycle^38,39,61^, and histidine degradation^42,43^ (**A**); fatty acid beta-oxidation networks and glycolysis^28,29,45,46^ (**B**); epilepsy and anxiety^49,51,52,54^ (**C**); and pheromones^34,50,56^ (**D**). Abbreviations: 2-acetyl-pyrroline, 2AP; 5-formiminotetrahydrofolate, 5-formimino-THF; 5-methyltetrahydrofolate, 5-MTHF, aldehyde dehydrogenase 7 family member A1, ALDHTA1; betaine aldehyde dehydrogenase, BADH; cytochrome C oxidase subunit II, COX-2; 3,4-dehydro-*exo*-brevicomin, DEB; disulfide, dimethyl, DSDM; dimethyl trisulfide, DMTS; formimidoyltransferase cyclodeaminase, FTCD; gamma-aminobutyric acid, GABA; glutamate decarboxylase, GAD; hydrogen sulfide, H_2_S; histidine ammonia-lyase, HAL; phosphatidylcholine, PC; trimethylamine, TMA; 2,3,5-trithiahexane, TTH; vitamin B6, vB6. The percentage sign, %, indicates that FTCD and vB6C have bifunctional activities. Enzymes and coenzymes are enclosed in squares. Blue and red arrows show the differential VOCs (in italics) after kindled seizures; pathways described by green lines, arrows, and squares are derived from non-mammalian organisms.

It is known that the components of VOCs that are excreted from the human body reflect the metabolic condition of the individual. It has been suggested that VOCs could be useful in the olfactory diagnosis of several disorders^23^, including infectious diseases, inherited disorders of metabolism, and lung cancer^24^. Brown and Goldstein^25^ proposed the presence of seizure-alertness in dogs, who might recognize pre-ictal human behavioural changes, changes in heart rate, and olfactory cues, while Corne *et al*.^26^ suggested that human prostate cancer could be detected by dogs sniffing the patient’s urine. Based on the suggestions of these reports, we hypothesized the possibility that urinary VOCs in human epilepsy might be detectable by olfactory cues. On the other hand, in previous our study with a TLE model using amygdala-kindled mice, the expression of growth hormone, which is, main hormone involved in lipid metabolism, was up-regulated along the neural circuits^27^. A ketogenic diet, high in fat, improves seizures^4,5^ in humans, suggesting that extracranial lipid metabolism is associated with convulsions seen in epileptic seizures. Hence, we formulated a hypothesis that the development of epilepsy is correlated with differences in organic compounds that originate from lipid metabolism, following development of epilepsy. As organic compounds that originate from lipid metabolism are highly volatile, we screened urinary VOCs in amygdala-kindled mice. We identified 15 types of VOCs, including two unknown-VOCs showing differential levels in the urine of amygdala-kindled mice, with AUCs above 0.8 (Table 2). Four VOCs, i.e., 2-buanone, 2-pentanone, and 2-heptanone of methyl ketones and 3,4-dehydro-*exo*-brevicomin are formed from fatty acids^28,29^ (Fig. 5B), showing differences following kindled-seizures. 2-butanone, 2-pentanone, and 3,4-dehydro-*exo*-brevicomin increased 1.31, 1.99, and 1.39 times, respectively, and 2-heptanone decreased 0.55 times. This suggests that TLE induced changes in lipid metabolism, resulting in the differential urinary VOCs. On the other hand, 4 VOCs including sulphur, 2 VOCs of nitrogen compounds, and aromatic VOCs including 2-acetyl-1-pyrroline, 2-acetylpyrrole, and acetophenone were also detected as biomarkers of TLE in mice (Fig. 3). This suggests that epileptic seizures also induced other metabolic changes.

Promax rotation and dendrogram analysis separated the 13 VOCs into 3 groups (Fig. 3C,D). VOCs were classified based on differential abundance between epileptic and sham-operated mice. VOCs with increased levels, such as 2-butanone, 2-pentanone, and 3,4-dehydro-exo-brevicomin, in kindled-mice were categorized to one group. Among the decreased VOCs, 2 pyrrole and 2 nitrogen compounds were categorized as the second and third groups, respectively. On the other hand, 4 VOCs including sulphur were divided to two groups, proposing that differential metabolic system works between methanthiol/2,3,5-trithiahexane and disulfide, dimethyl/dimethyl trisulfide in mice.

The findings of TLE-responsive urinary VOCs were based on SPME collection and GC-MS analyses. As we focused on urinary VOCs that originated from lipid metabolism, we first selected a divinylbenzene/carboxen/polydimethylsiloxane (DVB/CAR/PDMS) fibre, which is highly efficient for the collection of C3-C20 volatile and semi-volatile compounds. The extraction time was 60 min as described by Hanai *et al*.^24^. On the other hand, we used different temperature conditions (37 °C, 45 °C and 60 °C) below 60 °C, because of the possibility of degradation of the components at higher temperatures could inhibit the efficiency of the volatile VOCs. As a result, each TIC peak intensity increased in proportion to an increase in temperature. The number of TIC peaks were 109 at 37 °C, 143 at 45 °C, and 144 at 60 °C (Fig. S1). As there was little difference in the data obtained at 45 °C and 60 °C, we chose the data obtained at 45 °C.

Since we screened urinary VOCs in both amygdala kindling and sham operated-mice, in this study, we were unable to select an internal standard for each VOC for the SPME collection and GC-MS analysis. Hence, we determined the recovery rate of the SPME collection in C57Bl/6j urine (200 μL) including 100 ng of *p*-bromofluorobenzene (standard solution 021-12041, Wako Pure Chemical industries, Ltd.) against a liquid injection (1 μL) of 100 ng of *p*-bromofluorobenzene in GC-MS (Fig. S2). There was little difference of absolute area (*m/z* 174) between the liquid injection and SPME collection, as shown by the complete recovery by SPME collection (104%). We injected *p*-bromofluorobenzene collected by SPME at the start and the end of the multiple sample inspection using the multifunctional autosampler system AOC-6000 (162 wells for samples) in every GC-MS analysis and obtained the same absolute areas (*m/z* 174) between the start and end. Hence, the GC-MS was run once using the multifunctional autosampler system, the absolute area of each *m/z* in individual urine samples were analysed, and VOCs were compared semi-quantitatively, as shown in Table 1. Quantitative methods for urinary VOCs, which were determined as biomarkers for mice TLE, should be developed using internal standards, in the near future.

Next, we investigated the metabolic pathways to which the 13 VOCs are associated and how they might be associated to epilepsy and other phenotypes. First, the volatile sulfur compounds (VSCs) methanethiol; disulfide, dimethyl; dimethyl trisulfide; and 2,3,5-trithiahexane decreased in the urine of mice following kindled seizures. Hydrogen sulfide (H_2_S) and methanethiol are naturally formed in mammalian tissues^30,31^ and by microbiota in the intestinal tract^32^ (Fig. 5A). In mammalian tissues, Kolluru *et al*.^31^ summarized in a 2013 review that H_2_S can be produced by enzymatic and non-enzymatic pathways: Cystathionine gamma-lyase (CSE) in the vasculature and liver, cystathionine beta-synthase (CBS) in the brain, nervous system, and liver, and 3-mercaptopyruvate sulfurtransferase (3-MST) in the brain and vasculature function as enzymes; non-enzymatically, H_2_S is generated from glucose via glycolysis or from phosphogluconate via NADPH oxidase. In fact, glucose reacts with methionine, homocysteine and cysteine, leading to methanethiol and H_2_S. Additionally, methanethiol is also produced by thiol S-methyltransferase with H_2_S^30^ and oxidizes spontaneously to disulfide, dimethyl^33^. In the gut flora, the review by Martínez-Cuesta *et al*. in 2013^32^, focusing on lactic acid bacteria in cheese microbiota, states that methanethiol is normally derived from methionine in the presence of pyridoxal phosphate and oxidizes to dimethyl disulfide and dimethyl trisulfide. On the other hand, the metabolism of 2,3,5-tritiahexane in either mammalian tissues or gut flora remains unknown. However, a study showed that male mice release urinary 2,3,5-tritiahexane as a pheromone^34^ (Fig. 5C) and Spadone *et al*.^35^ demonstrated in 2006 that the thermal degradation products of methionine and photolysis of dimethyl trisulfide lead to the exogenous production of 2,3,5-tritiahexane. Hence, the endogenous metabolic systems related with the methionine-homocysteine cycle might affect polysulfuration. If so, decreased level of 2,3,5-tritiahexane might be observed following epileptic seizures as the decreased polysulfuration (Fig. 5A).

Second, 2-acetyl-1-pyrroline, 2-acetylpyrrole, and trimethylamine also decreased in mice urine following kindled seizures (Fig. 5A). The first two normally originate from food^36^, while trimethylamine comes from the gut flora^37^. 2-acetyl-1-pyrroline is the most important aroma compound in rice and can easily oxidize to 2-acetylpyrrole at room temperature, so that strong correlations between 2-acetyl-1-pyrroline and 2-acetylpyrrole determine the aromatic varieties of rice^36^. The phospholipid PC is the most significant dietary source of choline and the enzyme betaine aldehyde dehydrogenase (Badh2) metabolizes betaine-aldehyde to forms betaine via choline, and inhibits the biosynthesis of 2-acetyl-1-pyrroline^38^. On the other hand, mutations of the human gene ALDH7A1^39^, the homolog of Badh2, cause pyridoxine-dependent epilepsy^40^. In amygdala-kindled epileptic mice, activation of ALDH7A1 might lower the stability of 2-acetyl-1-pyrroline and 2-acetylpyrrole. It has been reported that microbiota metabolizes PC and choline to form trimethylamine^37^, which functions as a pheromone in both females and males^33,41^ (Fig. 5C).

Third, methane, nitro- also decreased in mice urine following kindled seizures (Fig. 5A). While methane, nitro- has not been detected in human blood, excretions such as urine and feces, or saliva, rats subcutaneously injected with methane, nitro- were used as a model of human histidinemia, showing increased levels of histidine in blood, urine, and cerebrospinal fluid, in which methane, nitro- is a histidine ammonia-lyase inhibitor (HAL)^42^. Furthermore, it is known that L-cysteine also inhibits HAL activity^43^. As the histidine degradation system includes the production of glutamate and GABA (Fig. 5A), modulation of HAL activity might affect the development and/or severity of seizures.

Fourth, 2-butanone and 2-pentanone increased and acetophenone and 2-heptanone decreased in the urine of mice following kindled seizures (Fig. 5B,C). These four VOCs have generally been described as flavor ingredients included in various foods and detected also in human excretory biospecimens, such as feces, saliva, and/or urine (Human Metabolome Database). Metabolically, while acetophenone is derived from L-phenylalanine in plants, such as *Camellia sinensis* (tea)^44^, 2-butanone, 2-pentanone, and 2-heptanone are methyl ketones derived by the fatty acid beta-oxidation network^45^ and glycolysis reactions in bacteria^28,46^ (Fig. 5B). Specifically, it was demonstrated that 2-butanone and 2-pentanone are produced from glucose by *Klebsiella pneumoniae* in gut flora^47^ and from hexanoic acids by *Penicillium roqueforti* in blue cheese^48^, respectively.

Associations of these VOCs with epilepsy and anxiety have been previously reported^49,50^ (Fig. 5C). Pettersson *et al*.^51^ showed that 2-pentanone reduced COX-2 protein levels in cultured cells originating from cancer, and Silva *et al*.^52^ showed that COX-2 mRNA and acetophenone decrease synchronously with β-glucan *in vitro*. COX-2 is an inducible enzyme expressed as an immediate early response gene, and is involved in the derivation of prostaglandins from arachidonic acid, and associated with inflammation and cancer. We have demonstrated that the kindling mice upregulated COX-2 expression in the brain immediately after 3 seizures as the onset of epilepsy^9^. On the other hand, the present amygdala-kindled mouse suffers from chronic temporal lobe epilepsy that causes daily seizures for about 40 days after the onset. Cavazos *et al*.^53^ observed neuronal loss in the CA3 field of hippocampal, entorhinal cortex, and the rostral endopyriform nucleus after 30 seizures. Currently, urine carrying the four VOCs was collected from chronic TLE mice suffering from 4 to 40 seizures, and not in the period immediately following onset. Therefore, it is suggested that urine during development of chronic TLE, accompanied with structural changes in the mesial temporal lobe, was collected. Hence, increase of 2-pentanone and decrease of acetophenone might occur as a negative feedback of epileptic seizures. Furthermore, reports have shown that 2-butanone blocked status epilepticus induced by lithium-pilocarpine in rats^49^, and 2-heptanone enhanced the neural activity of the basal amygdala accompanied by the removal of the anterior olfactory epithelial organs^54^. The urine of mice suffering from chronic TLE exhibited increased 2-butanone and decreases 2-heptanone, which might suggest a negative feedback of chronic seizures. Hence, when epilepsy progresses chronically, 2-butanone and 2-pentanone might be produced as seizure-suppressants, possibly accompanied by a decrease of acetophenone and 2-heptanone that are associated with neuronal activity.

Fifth, four of the VOCs identified as urinary biomarkers of mouse epilepsy, namely trimethylamine (TMA), 2,3,5-trithiahexane (TTH), 2-heptanone, and 3,4-dehydro-exo-brevicomin (DEB) are pheromones (Fig. 5D). Trimethylamine, 2,3,5-trithiahexane, and 2-heptanone, which have been detected in both mouse and human urine, decreased following mouse kindled-seizures, while DEB which has not been detected in human urine, increased. The excretion of trimethylamine in female mice and dimethylamine in women have been reported to be affected by diurnal rhythms^33^ and trimethylamine also functions as an attractant pheromone for mice and an aversive one for humans via trace amine-associated receptor 5 (TAAR5)^41^. 2,3,5-trithiahexane is excreted by males^34^ and a candidate human receptor was identified as OR2T11^55^, however its function is uncertain. The 2-heptanone excreted by males functions as an attractant pheromone to females and as an alarm one to males^50,54^. On the other hand, DEB is also a male pheromone attractant to females^56^ and seems to share similar pheromone activity with trimethylamine and 2-heptanone. Currently, the opposite tendencies of these pheromones, namely the increase of DEB and the decrease of trimethylamine and 2-heptanone, remain to be explained. However, Song *et al*.^29^ suggested that DEB is synthesized with omega-3-decenoyl-CoA, produced by a limited beta-oxidation with palmitoleic acid (16:1) and oleic acid (18:1), in the beetle body fat. On the other hand, we observed an increase of palmitoleic acid and oleic acid in the plasma of kindled-mice (manuscript in preparation), which might necessarily lead to a DEB increase.

Taken together, the urinary differential VOCs we identified suggest that TLE induced by amygdala stimulation could induce metabolic changes both endogenous and in the gut flora. Our preliminary observation was that there were few differences in the dietary content of the kindled and sham operated-mice, who were fed with the help of a metabolic cage (KN-645, Natsume Seisakusho, Tokyo, Japan), which was 3.4314 ± 0.2351 g in kindled-mice (average ± S.E.M., n = 7) and 3.5886 + 0.1795 in sham operated-mice (n = 7) (*p* = 0.8747 in Mann Whitney *U*-test, two-way). It is thought that repetitive seizures did not induce malnutrition, which might be associated with the changing gut flora. Thus, further investigations are needed into the TLE-induced changes in the gut flora. Moreover, we determined *m/z* areas of 15 VOCs in the diet (MF 12 mm φ pellet, Oriental Yeast Co. Ltd., Tokyo, Japan) (Table S2). Table S2B shows that 7 VOCs were detected in the diet from 15 VOCs, in which trimethylamine; methanthiol; 2-butanone; disulfide, dimethyl; 2-heptanone; dimethyl trisulfide; and 2-acetylpyrrole were found abundantly in the diet of the mice, suggesting both possibilities that the VOCs might be metabolized from those adsorbed from the diet and produced by the metabolic system. The other VOCs that are not found in the diet, might be metabolized endogenously and/or by the gut flora, such as 2-pentanone; methane, nitro-; RI1227; 2-acetyl-1-pyrroline; 3,4-dehydro-exo-brevicomin; RI1449; acetophenone; and 2,3,5-trithiahexane. In future research, we will take into account our VOC results in investigating possible correlations induced by TLE between peripheral metabolic system and brain function.

Epileptic seizures lead to alteration in the blood^57^. For example, the blood ammonia level increases in convulsions, inducing acidosis, leading to a medical emergency^58^. These are considered as epilepsy-responsive symptoms for two reasons: (1) elevation of blood ammonia level occurs along with extensive muscle contractions, resulting in acidosis, (2) cardiopulmonary arrest or haemorrhagic shock causes acidosis, resulting in ammonia production by the red blood cells, leading to hyperammonemia in patients^58^. Moreover, other blood products such as creatine, lactate, hormones including prolactin, and creatinine kinase, etc, are also detected postictally. However, these products are detected in both non-epileptic and epileptic seizures^57^. The metabolic changes induced by TLE still remain unexplained. We were unable to isolate VOCs related to hyperammonemia in the present study. We should investigate endogenous metabolism in the blood of mice with TLE induced by amygdala stimulation and the linkage of excreted urinary VOCs with endogenous blood metabolites.

In conclusion, mesial TLE includes foci in the amygdala, hippocampus and surrounding cortex and exhibits common symptoms in many mammals including humans. Many species of mammals have been used as experimental models of TLE, which can be induced by amygdala and hippocampus kindling stimulations to clarify the mechanisms of developmental TLE. The present results suggest that urinary VOCs, detected by SPME GC-MS, can potentially be metabolic biomarkers of TLE in mice. The hypothesis that altered urinary VOCs profiles may be derived from specific metabolic cascades could lead to the identification of common biomarkers for human and animals, and thus deserves further investigation. In particular, urine sampling could represent a simple and safe alternative to more invasive procedures in children and domestic animals.

## Methods

### Ethics statement

All animals were treated in accordance with the Guidelines for Proper Conduct of Animal Experiments published by the Science Council of Japan (2006). The protocol was approved by the Committee on the Ethics of Animal Experiments of the Kyoto Sangyo University (Approval No. 2017- 09, 2018-08).

### Preparation of kindled mice

Mice (8 weeks old, male; C57Bl/6j from CREA Japan, Inc, Tokyo) were housed for one week to recover from transportation stress. All surgical procedures were conducted under anesthetization with isoflurane (Pfizer, Tokyo, Japan) as described previously^22^. Briefly, a unipolar cathode electrode made of tungsten steel, 0.1 mm wide (Inter Medical co, Ltd., Nagoya, Japan), and an anode electrode, made of a screw, 1.0 mm wide and 3.0 mm long (Biotex Kyoto, Japan), were implanted on the right side of the basolateral amygdala (A -2.0, L 3.0, V 4.5 mm from the bregma) and on the left side of the subdural space (A 2.0, L 1.5 mm from the bregma), respectively. Ten days after surgery, unrestrained conscious mice (age 10 weeks) received a biphasic square wave pulse (480 μA; 60 Hz, 200 μs duration, for 2 s) using an electrical stimulator (SEN-3301, Nihon Kohden, Tokyo, Japan) and an isolator (SS-202J) once a day. Electroencephalographic (EEG) recordings of the subdural space were carried out with bilateral electrodes before and after stimulation using PreAmp and Head Amp (BEMCT-21 and BH-3, Low cut = 0.5, High cut = 30; Biotex, Kyoto, Japan) and the data acquisition program SleepSign, ver. 2.0 (Kissei Comtec co., ltd., Nagano, Japan). The number of spikes and the duration of the afterdischarges were manually calculated based on the recorded EEGs using SleepSign, ver. 2.0. Seizures were monitored according to the modified Racine’s criteria^22^. Urine was collected from full-kindled mice following more stimulation for 3 days until day 60 (18.5 weeks old), frozen quickly under liquid N_2_, and stored in nitrogen gas tanks until just before use. Sham-operated mice without electro-stimulation were used as controls.

Urinary creatinine concentrations were determined with the LabAssay^TM^ Creatinine colorimetry kit (Wako Pure Chemical industries, Ltd. Osaka, Japan), based on the Jaffe method^59^.

### Chemicals

Standard chemicals were used as follows: trimethylamine (25% pure methylamine, *N*, *N*,-dimethyl-, in ethanol, catalog No. T2892); 2-butanone (>99.0% (GC), No. E0140); 2-pentanone (>99.0% (GC), No. P0060); disulfide, dimethyl (>99.0% (GC), No. D0714); 2-heptanone (>98.0% (GC), No. H0037); dimethyl trisulfide (>98.0% (GC), No. D3418); butanoic acid, 3-methyl- (>99.0% (GC), No. M0182); acetophenone (>98.5% (GC), No. A0061); 2-acetylpyrrole (>98.0% (GC) ethanone, 1-(1H-pyrrol-2-yl)-, No. A0894); formamide, N-phenyl- (99%, No. F0047) from Tokyo Chemical Inc. (Tokyo, Japan). 3-penten, 2-one (70% pure, No. 145017, Sigma, MO, USA); 1-nitro-2-methyl propene (>98.0%, No. sc-481890, Santa Cruz, CA, USA); methanethiol (1 µg/µL benzene solution, No. 130-06173, FUJIFIUM Wako Pure Chemical Corp. Osaka, Japan); *n*-alkane mix solution (C9-C40: 50 µg/mL; C10, 20, 30, and 40: 100 µg/mL, No. 102158321, GL Sciences Inc., Tokyo, Japan).

### Solid-phase microextraction (SPME)

Urine extraction employed 50/30 µm divinylbenzene/carboxen/polydimethylsiloxane fibers (Supelco/Sigma-Aldrich, Bellefonte, PA, USA). The method involved exposing the SPME fiber, which had been inserted into the glass vial with 200 µL urine, to the gaseous sample for 60 min at the constant temperature of 45 °C. Then, the hot gas chromatography (GC) injector was used for de-adsorption of the volatile compounds, which proceeded with splitless pulse for 3 min at 240 °C.

### Gas chromatography - mass spectrometry (GCMS) analysis

A GC-MS (Shimadzu GCMS QP-2010 Ultra, Shimadzu Co., Kyoto, Japan) equipped with an InertCap Pure-WAX with ProGuard and T.L. column (60 m + 10 m pro-guard line and 2 m transfer line, 0.25 mm internal diameter, 0.5 µm film thick; GL Sciences Inc.) was used for sample analysis. The oven temperature was programmed as follows: 40 °C for 10 min, ramped to 240 °C at 5 °C min^−1^ and held for 10 min. Helium was used as carrier gas at a constant linear velocity of 20 cm/s. Operating parameters for the mass spectrometer were as follows: ion source temperature, 200 °C; ionizing energy, 70 eV; scanning frequency 0.2 s/spectrum from *m/z* range: 30 to 300; column length, 60 m.

Shimadzu GCMSsolution ver. 4.45 was used to convert raw GC-MS data into the mzXML format, and the XCMS software package, ver. 1.3.2 (http://masspec.scripps.edu), running under R, version 3.2.3 (http://cran.r-project.org/), was used for extracting differential ion peaks (*m/z*) between kindled and sham-operated mice. Total ion currents (TICs) of 24 metabolites were extracted based on the retention times of the ion peaks associated to *p* < 0.05 according to the GCMSsolution software (Table 1). Metabolites in the TICs were tentatively identified by searching mass spectra in the NIST/EPA/NIH mass spectral library (NIST14). Furthermore, identification of each metabolite was achieved by comparing the fragmentation patterns of ion peaks and the retention times with standards. Additionally, the identification was also accomplished by matching their retention indices (RI), calculated in relation to the retention time of n-alkanes series (Kovats indices, 1958), with the data in NIST Chemistry WebBook^60^. We used the multifunctional autosampler system AOC-6000 (Shimadzu Co.) for stable measurement of all samples. Metabolite concentration was determined by calculating the ratio of the ion peak area of volatiles and the peak area of the limiting diluted external standard.

### Statistical analyses

The absolute area of each ion peak was indicated as mean ± standard error of the mean (S.E.M.), and compared between the two groups using the Mann-Whitney *U*-test (Table 1), with *p*-values ≤ 0.05 considered as statistically significant. To determine the ability of each VOC to separate epileptic mice from sham-operated mice, receiver operating characteristic (ROC) curves were constructed, which plot “sensitivity” against “1 - specificity” (GraphPad Prism 6.07 for Windows, GraphPad Software, La Jolla, CA, USA). The area under the ROC curve (AUC) was calculated to estimate the predictive power of these potential biomarkers in distinguishing epilepsy from sham-operated controls.

In parallel, exploratory data analysis using the volatile compounds showing significant differences between the groups was performed by principal component analysis (PCA) (IBM SPSS Statistics 25), to validate the predicted probabilities of belonging to each group. During autoscaling, principal components were extracted with absolute values of VOCs in the “*Factor Analysis*” menu of the SPSS, in which “*Correlation matrix*” in analysis, “*Unrotated factor solution*” in display and six factors in extraction were selected as the condition of PCA. The principal factor scores for individuals (variables) and the principal factor score coefficient matrix were saved using the regression method. Additionally, VOCs were grouped based on their factor loadings resulting from promax rotation with Kaiser normalization, and using a dendrogram built with the Ward method from the unrotated loadings of the first six components in the component matrix extracted using PCA without rotation. Finally, stepwise linear discriminant analysis was performed (IBM SPSS Statistics 25), as the Box’s M test suggested homogeneity of covariance matrices (F (6, 2540) = 0.207, *p* = 0.975).

## Supplementary information

Supplementary Information

## Data Availability

All data generated or analyzed during this study are included in this published article and its Supplementary Information files.
