# Supplementary material for: Urinary volatile metabolites of amygdala-kindled mice reveal novel biomarkers associated with temporal lobe epilepsy
Source: Sci Rep. 2019 Jul 22;9:10586. doi: 10.1038/s41598-019-46373-8 (PMC6646363; doi:10.1038/s41598-019-46373-8)
Supplement: Supplementary file 1 — Supplementary Information [file 41598_2019_46373_MOESM1_ESM.doc]

**Supplementary Information**

Title: Urinary volatile metabolites of amygdala-kindled mice reveal novel biomarkers associated with temporal lobe epilepsy

Akiko Fujita, Manami Ota, and Keiko Kato*

Faculty of Life Sciences, Kyoto Sangyo University, Motoyama, Kamigamo, Kita-ku, Kyoto, Japan

*Corresponding author

**Running title:** Urinary metabolites and epilepsy

**Keywords:** amygdala, epilepsy, urine, volatile organic compounds, biomarker

**Supplementary Table1.** Volatile organic compound (VOC) names, similarity index, chemical formula, CAS No. and molecular weight analyzed by GC-MS using InertCap PureWAX column under the operating parameters detailed in the *Methods*.

| **No** | **RT 1** | **RT 2** | **SI** | **VOCs** | **Chemical Formula** | **CAS** | **MW** |
| --- | --- | --- | --- | --- | --- | --- | --- |
| 1 | 5.22 | 7.72 | 96 | Carbamic acid, monoammonium salt | CH6N2O2 | 1111-78-0 | 78 |
| 2 | 5.18 | 7.77 | 93 | Carbon dioxide | CO2 | 124-38-9 | 44 |
| 3 |  | 7.77 | 86 | dl-Alanyl-l-alanine | C6H12N2O3 | 59247-16-4 | 160 |
| 4 | 5.19 |  | 95 | Nitrous oxide | N2O | 10024-97-2 | 44 |
| 5 | 5.27 |  | 93 | Ethyne, fluoro- | C2HF | 2713/9/9 | 44 |
| 6 |  | 8.54 | 89 | Oxirane, 2,3-diethyl- | C6H12O | 4468-66-0 | 100 |
| 7 | 6.15 | 8.58 | 97 | Trimethylamine | C3H9N | 75-50-3 | 59 |
| 8 |  | 8.86 | 89 | 1-Pentene | C5H10 | 109-67-1 | 70 |
| 9 |  | 8.87 | 97 | Cyclopentane | C5H10 | 287-92-3 | 70 |
| 10 |  | 10.18 | 93 | Cyclohexane | C6H12 | 110-82-7 | 84 |
| 11 |  | 11.85 | 85 | Propanal | C3H6O | 123-38-6 | 58 |
| 12 |  | 12.67 | 96 | Acetone | C3H6O | 67-64-1 | 58 |
| 13 |  | 14.98 | 87 | 1,5-Dimethyl-6-oxa-bicyclo[3.1.0]hexane | C7H12O | 82461-31-2 | 112 |
| 14 |  | 15.00 | 90 | 3-Hexen-2-one, 5-methyl- | C7H12O | 5166-53-0 | 112 |
| 15 |  | 15.75 | 86 | Furan, 2-methyl- | C5H6O | 534-22-5 | 82 |
| 16 | 13.61 | 15.99 | 95 | 2-Butanone | C4H8O | 78-93-3 | 72 |
| 17 | 14.24 | 16.59 | 92 | Butanal, 2-methyl- | C5H10O | 96-17-3 | 86 |
| 18 |  | 16.70 | 91 | Butanal, 3-methyl- | C5H10O | 590-86-3 | 86 |
| 19 |  | 17.15 | 97 | Isopropyl Alcohol | C3H8O | 67-63-0 | 60 |
| 20 | 15.14 |  | 91 | 2-Butanone, 3-methyl- | C5H10O | 563-80-4 | 86 |
| 21 | 15.16 | 17.40 | 90 | Ethanol | C2H6O | 64-17-5 | 46 |
| 22 | 15.86 |  | 85 | 4-Octen-3-one, 6-ethyl-7-hydroxy- | C10H18O2 | 0-00-0 | 170 |
| 23 |  | 18.01 | 85 | 2-Hexenal, 2-ethyl- | C8H14O | 645-62-5 | 126 |
| 24 |  | 18.87 | 91 | Heptane, 2,2,4,6,6-pentamethyl- | C12H26 | 13475-82-6 | 170 |
| 25 |  | 18.89 | 93 | Decane, 2,2-dimethyl- | C12H26 | 17302-37-3 | 170 |
| 26 | 17.23 | 19.42 | 96 | 2-Pentanone | C5H10O | 107-87-9 | 86 |
| 27 | 18.51 |  | 87 | Methyl Isobutyl Ketone | C6H12O | 108-10-1 | 100 |
| 28 |  | 21.79 | 87 | Ethanone, 1-(2-furanyl)- | C6H6O2 | 1192-62-7 | 110 |
| 29 |  | 21.98 | 91 | 3-Buten-2-ol, 2-methyl- | C5H10O | 115-18-4 | 86 |
| 30 |  | 22.08 | 88 | Heptane, 5-ethyl-2,2,3-trimethyl- | C12H26 | 62199-06-8 | 170 |
| 31 |  | 22.72 | 91 | 2,3-Pentanedione | C5H8O2 | 600-14-6 | 100 |
| 32 |  | 22.88 | 86 | 1-Hexene, 4-methyl- | C7H14 | 3769-23-1 | 98 |
| 33 |  | 23.43 | 85 | 2-Pentenal, 2,4,4-trimethyl- | C8H14O | 53907-61-2 | 126 |
| 34 |  | 23.60 | 93 | Disulfide, dimethyl | C2H6S2 | 624-92-0 | 94 |
| 35 |  | 23.80 | 88 | 2-Hexanone | C6H12O | 591-78-6 | 100 |
| 36 |  | 23.83 | 89 | Hexanal | C6H12O | 66-25-1 | 100 |
| 37 |  | 23.93 | 88 | 2-Cyclopenten-1-one, 3,4,4-trimethyl- | C8H12O | 30434-65-2 | 124 |
| 38 |  | 23.98 | 87 | 5-Octyn-4-one, 2,2,7,7-tetramethyl- | C12H20O | 28884-89-1 | 180 |
| 39 |  | 24.00 | 87 | 6-Methyl-3,5-heptadiene-2-one | C8H12O | 1604-28-0 | 124 |
| 40 | 20.57 |  | 86 | 3-Hexanone | C6H12O | 589-38-8 | 100 |
| 41 | 20.90 |  | 86 | Hexane, 4-ethyl-2-methyl- | C9H20 | 3074-75-7 | 128 |
| 42 | 21.85 |  | 85 | 2-Propenoic acid, 1,4-butanediyl ester | C10H14O4 | 1070-70-8 | 198 |
| 43 | 21.87 | 24.11 | 92 | Butanenitrile, 2-methyl- | C5H9N | 18936-17-9 | 83 |
| 44 |  | 24.42 | 85 | 2-Pentenal, (E)- | C5H8O | 1576-87-0 | 84 |
| 45 |  | 24.43 | 86 | 2-Butenal, 2-methyl-, (E)- | C5H8O | 497-03-0 | 84 |
| 46 |  | 25.09 | 96 | (S)-(+)-2-Pentanol | C5H12O | 26184-62-3 | 88 |
| 47 |  | 25.12 | 88 | 2-Heptanol, 3-methyl- | C8H18O | 31367-46-1 | 130 |
| 48 |  | 25.15 | 91 | 2-Pentanol | C5H12O | 6032-29-7 | 88 |
| 49 |  | 25.56 | 90 | 2,3-Hexanedione | C6H10O2 | 3848-24-6 | 114 |
| 50 | 23.05 |  | 91 | (R)-(-)-2-Pentanol | C5H12O | 31087-44-2 | 88 |
| 51 | 23.42 | 25.64 | 93 | 3-Penten-2-one | C5H8O | 625-33-2 | 84 |
| 52 | 23.44 |  | 93 | Ethanone, 1-cyclopropyl- | C5H8O | 765-43-5 | 84 |
| 53 | 23.89 | 26.01 | 91 | 1-Butanol | C4H10O | 71-36-3 | 74 |
| 54 |  | 26.29 | 88 | Benzene, 1,3-dimethyl- | C8H10 | 108-38-3 | 106 |
| 55 | 23.58 | 26.33 | 86 | o-Xylene | C8H10 | 95-47-6 | 106 |
| 56 |  | 26.51 | 94 | Methane, nitro- | CH3NO2 | 75-52-5 | 61 |
| 57 | 25.56 | 27.69 | 90 | 2-Hexanone, 5-methyl- | C7H14O | 110-12-3 | 114 |
| 58 | 25.63 | 27.70 | 89 | 2-Heptanone | C7H14O | 110-43-0 | 114 |
| 59 |  | 27.78 | 87 | Allyl acetate | C5H8O2 | 591-87-7 | 100 |
| 60 |  | 28.04 | 88 | Cyclobutylamine | C4H9N | 2516-34-9 | 71 |
| 61 |  | 28.05 | 93 | Hydrogen azide | HN3 | 7782-79-8 | 43 |
| 62 |  | 28.08 | 93 | 1-Tetrazol-2-ylethanone | C3H4N4O | 51410-11-8 | 112 |
| 63 |  | 28.15 | 86 | Acetic acid, (acetyloxy)- | C4H6O4 | 13831-30-6 | 118 |
| 64 |  | 28.28 | 90 | 2-Butenal, 3-methyl- | C5H8O | 107-86-8 | 84 |
| 65 |  | 28.38 | 86 | Butane, 2,2-dimethyl- | C6H14 | 75-83-2 | 86 |
| 66 |  | 28.60 | 85 | Limonene | C10H16 | 138-86-3 | 136 |
| 67 |  | 28.69 | 85 | Cyclohexanol, 1-methyl-4-(1-methylethenyl)-, acetate | C12H20O2 | 10198-23-9 | 196 |
| 68 | 26.66 | 28.84 | 93 | 3-Heptanone, 6-methyl- | C8H16O | 624-42-0 | 128 |
| 69 | 26.96 | 29.08 | 96 | 2-Penten-1-ol, acetate, (Z)- | C7H12O2 | 42125-10-0 | 128 |
| 70 | 26.77 | 29.13 | 95 | Cyclopentanone, 3-methyl- | C6H10O | 1757-42-2 | 98 |
| 71 |  | 29.78 | 89 | Propane, 1,1'-sulfonylbis- | C6H14O2S | 598-03-8 | 150 |
| 72 | 27.59 |  | 88 | 5-Hexen-2-one, 5-methyl- | C7H12O | 3240/9/3 | 112 |
| 73 | 27.60 | 29.83 | 89 | 4-Hepten-2-one, (E)- | C7H12O | 36678-43-0 | 112 |
| 74 | 27.88 | 30.14 | 90 | 5-Oxohexanenitrile | C6H9NO | 10412-98-3 | 111 |
| 75 |  | 30.38 | 87 | Hexadecane, 1-iodo- | C16H33I | 544-77-4 | 352 |
| 76 |  | 30.38 | 87 | Nonane, 5-(1-methylpropyl)- | C13H28 | 62185-54-0 | 184 |
| 77 |  | 30.39 | 89 | Nonane, 5-butyl- | C13H28 | 17312-63-9 | 184 |
| 78 |  | 30.39 | 86 | Dodecane, 4,6-dimethyl- | C14H30 | 61141-72-8 | 198 |
| 79 |  | 30.42 | 87 | Nonadecane | C19H40 | 629-92-5 | 268 |
| 80 |  | 30.43 | 86 | Sulfurous acid, hexyl octyl ester | C14H30O3S | 0-00-0 | 278 |
| 81 |  | 30.82 | 88 | 4,5-Octanedione | C8H14O2 | 5455-24-3 | 142 |
| 82 |  | 30.84 | 86 | Vinyl butyrate | C6H10O2 | 123-20-6 | 114 |
| 83 |  | 31.30 | 96 | Acetoin | C4H8O2 | 513-86-0 | 88 |
| 84 |  | 31.71 | 85 | 3,4,4,-Trimethyl-1-pentyn-3-ol | C8H14O | 993-53-3 | 126 |
| 85 | 28.27 |  | 90 | Styrene | C8H8 | 100-42-5 | 104 |
| 86 | 29.54 |  | 89 | Pentane, 2,2,4-trimethyl-4-nitro- | C8H17NO2 | 5342-78-9 | 159 |
| 87 | 29.54 |  | 86 | 2-Pentene, 2,3,4-trimethyl- | C8H16 | 565-77-5 | 112 |
| 88 | 29.55 |  | 89 | Pentane, 2-isocyano-2,4,4-trimethyl- | C9H17N | 14542-93-9 | 139 |
| 89 | 29.60 |  | 89 | 1-Hexene, 5,5-dimethyl- | C8H16 | 7116-86-1 | 112 |
| 90 | 30.07 | 32.42 | 93 | Pentane, 1-nitro- | C5H11NO2 | 628-05-7 | 117 |
| 91 | 30.14 | 32.42 | 93 | Pentane, 2-nitro- | C5H11NO2 | 4609-89-6 | 117 |
| 92 | 29.59 | 32.66 | 89 | 6-Hepten-3-one, 4-methyl- | C8H14O | 26118-97-8 | 126 |
| 93 | 30.38 |  | 90 | 3-Heptanone, 5-methylene- | C8H14O | 20690-70-4 | 126 |
| 94 | 30.85 | 33.15 | 86 | 2-Acetyl-1-pyrroline | C6H9NO | 85213-22-5 | 111 |
| 95 |  | 33.80 | 87 | 2-Methyl-pyrrolidine-2-carboxylic acid | C6H11NO2 | 42856-71-3 | 129 |
| 96 |  | 33.83 | 90 | 2-Pyrrolidinemethanol, 1-methyl- | C6H13NO | 3554-65-2 | 115 |
| 97 | 32.70 |  | 87 | 1-Nitro-2-methyl propene | C4H7NO2 | 1606-30-0 | 101 |
| 98 | 32.90 | 35.30 | 88 | 3,4-dehydro-*exo*-brevicomin | C9H14O2 | 62255-25-8 | 154 |
| 99 |  | 35.74 | 93 | Acetic acid | C2H4O2 | 64-19-7 | 60 |
| 100 | 33.73 | 36.10 | 90 | 2-Butene, 1-bromo-3-methyl- | C5H9Br | 870-63-3 | 148 |
| 101 | 33.74 | 36.17 | 89 | 3-Methyl-3-nitrobut-1-ene | C5H9NO2 | 1809-67-2 | 115 |
| 102 |  | 36.48 | 85 | Pyridine, 2,3,4,5-tetrahydro- | C5H9N | 505-18-0 | 83 |
| 103 |  | 38.46 | 86 | Linalool | C10H18O | 78-70-6 | 154 |
| 104 | 34.12 |  | 85 | 2,2-Dimethyl-3-hydroxypropionaldehyde | C5H10O2 | 597-31-9 | 102 |
| 105 | 34.13 |  | 86 | Oxetane, 2-methyl-4-propyl- | C7H14O | 7045-79-6 | 114 |
| 106 | 36.11 | 38.70 | 96 | Benzaldehyde | C7H6O | 100-52-7 | 106 |
| 107 |  | 38.98 | 85 | Propanoic acid, 2-methyl- | C4H8O2 | 79-31-2 | 88 |
| 108 |  | 39.21 | 90 | Propanoic acid, 2,2-dimethyl- | C5H10O2 | 75-98-9 | 102 |
| 109 | 39.15 | 41.61 | 93 | Butanoic acid, 3-methyl- | C5H10O2 | 503-74-2 | 102 |
| 110 |  | 42.04 | 92 | Acetophenone | C8H8O | 98-86-2 | 120 |
| 111 |  | 42.49 | 87 | 2,3,5-Trithiahexane | C3H8S3 | 42474-44-2 | 140 |
| 112 |  | 45.37 | 97 | o-Toluidine | C7H9N | 95-53-4 | 107 |
| 113 |  | 45.44 | 97 | Benzenamine, 3-methyl- | C7H9N | 108-44-1 | 107 |
| 114 | 39.51 |  | 91 | (E)-.beta.-Famesene | C15H24 | 18794-84-8 | 204 |
| 115 | 40.69 |  | 88 | Pentanoic acid | C5H10O2 | 109-52-4 | 102 |
| 116 | 42.79 |  | 89 | p-Aminotoluene | C7H9N | 106-49-0 | 107 |
| 117 | 43.14 | 45.75 | 97 | Hexanoic acid | C6H12O2 | 142-62-1 | 116 |
| 118 |  | 46.32 | 86 | 3,6,9,12-Tetraoxatetradecan-1-ol | C10H22O5 | 5650-20-4 | 222 |
| 119 | 44.04 | 46.75 | 86 | Propanoic acid, 2-methyl-, 3-hydroxy-2,2,4-trimethylpentyl ester | C12H24O3 | 77-68-9 | 216 |
| 120 |  | 47.40 | 85 | 2,2,4-Trimethyl-1,3-pentanediol diisobutyrate | C16H30O4 | 6846-50-0 | 286 |
| 121 | 44.79 | 47.52 | 93 | Dimethyl sulfone | C2H6O2S | 67-71-0 | 94 |
| 122 | 45.45 |  | 87 | Heptanoic acid | C7H14O2 | 111-14-8 | 130 |
| 123 | 45.49 | 48.09 | 92 | Hexanoic acid, 2-ethyl- | C8H16O2 | 149-57-5 | 144 |
| 124 | 46.23 | 48.95 | 93 | 2-acetylpyrrole | C6H7NO | 1072-83-9 | 109 |
| 125 | 47.73 |  | 87 | Octanoic acid | C8H16O2 | 124-07-2 | 144 |
| 126 |  | 49.73 | 96 | Heneicosane | C21H44 | 629-94-7 | 296 |
| 127 | 50.50 | 53.69 | 90 | Formamide, N-phenyl- | C7H7NO | 103-70-8 | 121 |
| 128 | 52.94 |  | 93 | Octacosyl acetate | C30H60O2 | 18206-97-8 | 452 |
| 129 | 52.94 |  | 93 | Acetic acid n-octadecyl ester | C20H40O2 | 822-23-1 | 312 |
| 130 | 53.00 |  | 91 | Eicosyl acetate | C22H44O2 | 822-24-2 | 340 |
| 131 | 54.52 |  | 85 | 1-Tetradecanol | C14H30O | 112-72-1 | 214 |
| 132 |  | 56.52 | 91 | Heptadecyl acetate | C19H38O2 | 822-20-8 | 298 |
| 133 |  | 56.61 | 87 | 1-Pentadecanol acetate | C17H34O2 | 629-58-3 | 270 |
| 134 |  | 57.16 | 91 | Pentacosane | C25H52 | 629-99-2 | 352 |
| 135 |  | 58.10 | 86 | Tetracontane | C40H82 | 4181-95-7 | 562 |

The RT 1 and RT 2 columns show the retention times in GC-MS with Shimadzu TQ-8040 and QP-2010 ultra, respectively.

**Supplementary Table 2. Composition and abundance of volatile organic compounds (VOCs) in MF diet**

**A. Composition of diet**

| General constituents |  | | |  |  |
| --- | --- | --- | --- | --- | --- |
| Water |  | | | 7.9 | g |
| Organic substances | Crude protein | | | 23.1 | g |
| ­ | Crude lipid | | | 5.1 | g |
|  | Crude fiber | | | 2.8 | g |
|  | Soluble  nitrogen-free extract | | | 55.3 | g |
| Inorganic substances | Crude ash | | | 5.8 | g |
| Calories |  | | | 359 | kcal |
| Vitamin | Vitamin A | | | 1283 | IU |
|  | Vitamin D3 | | | 137 | IU |
|  | Vitamin E | | | 9.1 | mg |
|  | Vitamin K3 | | | 0.04 | mg |
|  | VitaminB1 | | | 2.05 | mg |
|  | Vitamin B2 | | | 1.1 | mg |
|  | Vitamin C | | | 4 | mg |
|  | Vitamin B6 | | | 0.87 | mg |
|  | Vitamin B12 | | | 5.5 | µg |
|  | Inositol | | | 439 | mg |
|  | Biotin | | | 27 | µg |
|  | Pantothenic acid | | | 2.45 | mg |
|  | Niacin | | | 10.61 | mg |
|  | Colin | | | 0.18 | g |
|  | Folic acid | | | 0.17 | mg |
| Mineral | Calcium | | | 1.07 | g |
|  | Phosphorus | | | 0.83 | g |
|  | Magnesium | | | 0.24 | g |
|  | Sodium | | | 0.19 | g |
|  | Potassium | | | 0.9 | g |
|  | Iron | | | 10.6 | mg |
|  | Aluminum | | | 2.1 | mg |
|  | Copper | | | 0.78 | mg |
|  | Zinc | | | 4.89 | mg |
|  | Cobalt | | | 0.1 | mg |
|  | Manganese | | | 4.84 | mg |
| Amino acid | Ile | | | 0.89 | g |
|  | Leu | | | 1.74 | g |
|  | Lys | | | 1.24 | g |
|  | Met | | | 0.44 | g |
|  | Cys | | | 0.36 | g |
|  | Phe | 1.04 | | | g |
|  | Tyr | 0.68 | | | g |
|  | Thr | 0.89 | | | g |
|  | Trp | 0.28 | | | g |
|  | Val | 1.08 | | | g |
|  | Arg | 1.42 | | | g |
|  | His | 0.6 | | | g |
|  | Ala | 1.2 | | | g |
|  | Asp | 2.14 | | | g |
|  | Glu | 3.99 | | | g |
|  | Gly | 1.18 | | | g |
|  | Pro | 1.31 | | | g |
|  | Ser | 1.11 | | | g |
|  | Asn | n.d. | | | g |
|  | Gln | n.d. | | | g |
| Fatty acid | 14:0 | 0.5 | | | % |
|  | 16:0 | 15.9 | | | % |
|  | 16:1 (n-7) | 0.9 | | | % |
|  | 17:0 | nd | | | % |
|  | 18:0 | 2.6 | | | % |
|  | 18:1 (n-9) | 23.4 | | | % |
|  | 18:1 (n-7) | 1.4 | | | % |
|  | 18:1 (n-5) | n.d. | | | % |
|  | 18:2 (n-6) | 44.7 | | | % |
|  | 18:3 (n-3) | 3.7 | | | % |
|  | 18:4 (n-3) | 0.2 | | | % |
|  | 20:0 | 0.4 | | | % |
|  | 20:1 (n-11) | 0.8 | | | % |
|  | 20:1 (n-9) | 0.7 | | | % |
|  | 20:4 (n-6) | n.d. | | | % |
|  | 20:5 (n-3) | 1.0 | | | % |
|  | 22:0 | 0.3 | | | % |
|  | 22:1 (n-11) | 0.9 | | | % |
|  | 22:1 (n-9) | 0.1 | | | % |
|  | 22:5 (n-3) | 0.1 | | | % |
|  | 22:6 (n-3) | 1.5 | | | % |
|  | 24:0 | 0.3 | | | % |
|  | 24:1 (n-9) | 0.2 | | | % |
|  | Undetectable fatty acids | | 0.5 | | % |

n.d. not detected.

Numerical values were averages in MF diets (100 g) between January, 2007 to December, 2009 (provided by Oriental Yeast Co., Ltd.)

**B. Abundance of VOCs in MF diet**

|  |  | | | 0.1 g MF diet (n = 3) | |  |
| --- | --- | --- | --- | --- | --- | --- |
| NO^a)^ | Compound | RT (min) | *m/z* | Average (x10^3^) | S.E.M. (x10^3^) | Relative values^b)^ |
| 1 | Trimethylamine | 8.57 | 58 | 11646 | 3791.5 | 0.5400** |
| 2 | Methanethiol | 9.309 | 47 | 44.024 | 8.0025 | 0.6162** |
| 3 | 2-Butanone | 15.917 | 43 | 164.84 | 24.641 | 1.3098* |
| 4 | 2-Pentanone | 19.332 | 43 | n.d. | n.d. | 1.9853** |
| 5 | Disulfide, dimethyl | 23.494 | 94 | 25.415 | 1.3931 | 0.5395*** |
| 6 | 3-Penten, 2-one | 25.63 | 69 | n.d. | n.d. | n.s. |
| 7 | RI1148f) | 26.38 | 57 | n.d. | n.d. | n.s. |
| 8 | Methane, nitro- | 26.432 | 30 | n.d. | n.d. | 0.6607** |
| 9 | 2-Heptanone | 27.71 | 43 | 115.93 | 30.559 | 0.5543** |
| 10 | RI1213 | 29.059 | 69 | 24.187 | 3.3796 | n.s. |
| 11 | RI1227 | 29.85 | 43 | n.d. | n.d. | 0.3093*** |
| 12 | RI1237 | 30.06 | 43 | n.d. | n.d. | n.s. |
| 13 | RI1291 | 31.79 | 57 | n.d. | n.d. | n.s. |
| 14 | RI1310 | 32.400 | 43 | n.d. | n.d. | n.s. |
| 15 | 2-Acetyl-1-pyrroline | 33.15 | 43 | n.d. | n.d. | 0.6290** |
| 16 | Dimethyl trisulfide | 34.89 | 126 | 2.5273 | 0.13605 | 0.5121* |
| 17 | 1-Nitro-2-methyl propene | 35.14 | 39 | n.d. | n.d. | n.s. |
| 18 | 3,4-dehydro-exo-brevicomin | 35.29 | 43 | n.d. | n.d. | 1.3886* |
| 19 | RI1449 | 36.500 | 55 | n.d. | n.d. | 0.6380** |
| 20 | Butanoic acid, 3-methyl- | 41.551 | 60 | 431.42 | 58.631 | n.s. |
| 21 | Acetophenone | 42.04 | 105 | n.d. | n.d. | 0.5489** |
| 22 | 2,3,5-Trithiahexane | 42.49 | 61 | n.d. | n.d. | 0.2300*** |
| 23 | 2-acetylpyrrole | 48.95 | 94 | 11.470 | 0.64440 | 0.6947** |
| 24 | Formamide, N-phenyl- | 53.77 | 93 | n.d. | n.d. | n.s. |

The 0.1 g powder of MF diet within GC-vial (1.5 ml) was applied to SPME collection under the same condition as the SPME collection and GC-MS for urinary VOCs.

^a)^ Number and compound described in Table 1.

^b)^ Relative values of kindling mice against sham operated-mice and stars of *p*-values stars as described in Table 1.

n.s. "not significant"

n.d. "Compound was not detected"

**Supplementary Figure 1. Typical TIC of urinary VOCs in C57Bl/6J mice, extracted by SPME at 37^o^C (A), 45^o^C (B) and 60^o^C (C).**

The TICs were obtained from the analysis of the gaseous sample in the glass vial with urine (200 µL) by SPME (DVB /CAR / PDMS, 50 / 30 µm, 2 cm) and GC-MS equipped with an InertCap Pure-WAX column (60 m + 10 m pro-guard line and 2 m transfer line, 0.25 mm i.d., 0.5 um thick). The extraction time was 60 min. Desorption was performed at 240^o^C for 10 min. The injection was performed with splitless pulses (closed for 3 min). Temperature programming consisted of an initial temperature of 40^o^C for 10 min, followed by an increase of 5^o^C / min to 240^o^C, and a 10 min hold at the final temperature.

**Supplementary Figure 2. Typical TIC of 100 ng *p*-bromofluorobenzene analyzed by SPME in C57Bl/6J mice urine (A) and injection of 1 µL methanol solution (B).**

100 ng of *p*-bromofluorobenzene was added to 200 µL of urine and extracted by SPME as mentioned in the Method section. *p*-bromofluorobenzene was detected at 33.3 min. Absolute areas were 4.604 x 10^6^ for A and 4.419 x 10^6^ for B. The ratio of SPME to injection of methanol solution was 1.04.
